# Novel Lipid Biomarkers of Chronic Kidney Disease of Unknown Etiology Based on Urinary Small Extracellular Vesicles: A Pilot Study of Sugar Cane Workers

**DOI:** 10.3390/metabo15080523

**Published:** 2025-08-02

**Authors:** Jie Zhou, Kevin J. Kroll, Jaime Butler-Dawson, Lyndsay Krisher, Abdel A. Alli, Chris Vulpe, Nancy D. Denslow

**Affiliations:** 1 Department of Physiological Sciences and Center for Environmental and Human Toxicology, University of Florida, Gainesville, FL 32611, USA; jiezhou68@ufl.edu (J.Z.); krollk@ufl.edu (K.J.K.); cvulpe@ufl.edu (C.V.); 2 Department of Environmental and Occupational Health, Colorado School of Public Health, University of Colorado Anschutz Campus, Aurora, CO 80045, USA; jaime.butler-dawson@cuanschutz.edu (J.B.-D.); lyndsay.krisher@cuanschutz.edu (L.K.); 3 Department of Medicine, Division of Nephrology, Hypertension, and Renal Transplantation, University of Florida College of Medicine, Gainesville, FL 32610, USA; aalli@ufl.edu; 4 Department of Physiology and Aging, University of Florida College of Medicine, Gainesville, FL 32610, USA

**Keywords:** CKDu, biomarkers, lipidomics, small extracellular vesicles

## Abstract

Background/Objectives: Chronic kidney disease of unknown etiology (CKDu) disproportionately affects young male agricultural workers who are otherwise healthy. There is a scarcity of biomarkers for early detection of this type of kidney disease. We hypothesized that small extracellular vesicles (sEVs) released into urine may provide novel biomarkers. Methods: We obtained two urine samples at the start and the end of a workday in the fields from a limited set of workers with and without kidney impairment. Isolated sEVs were characterized for size, surface marker expression, and purity and, subsequently, their lipid composition was determined by mass spectrometry. Results: The number of particles per ml of urine normalized to osmolality and the size variance were larger in workers with possible CKDu than in control workers. Surface markers CD9, CD63, and CD81 are characteristic of sEVs and a second set of surface markers suggested the kidney as the origin. Differential expression of CD25 and CD45 suggested early inflammation in CKDu workers. Of the twenty-one lipids differentially expressed, several were bioactive, suggesting that they may have essential functions. Remarkably, fourteen of the lipids showed intermediate expression values in sEVs from healthy individuals with acute creatinine increases after a day of work. Conclusions: We identified twenty-one possible lipid biomarkers in sEVs isolated from urine that may be able to distinguish agricultural workers with early onset of CKDu. Differentially expressed surface proteins in these sEVs suggested early-stage inflammation. This pilot study was limited in the number of workers evaluated, but the approach should be further evaluated in a larger population.

## 1. Introduction

Chronic kidney disease of unknown etiology (CKDu), also known as Mesoamerican Nephropathy, affects agricultural workers in hot, humid areas around the world, especially those working on sugar cane plantations [1,2,3]. This disease predominantly affects young men (<40 years of age) who do not have hypertension or diabetes [4]. Mesoamerican agricultural workers are at a higher risk of developing CKDu than workers who have never worked in agriculture [4,5,6,7,8]. There are several conditions that could lead to increased stress on the kidneys in these populations, including inadequate fluid ingestion, working in excessively hot and humid conditions, the use of nonsteroidal anti-inflammatory drugs (NSAIDS) [9], and exposure to environmental contaminants such as pesticides [10] and silica particles from sugar cane ash [11] during the harvesting process. This condition is not limited to sugar cane plantations as it has been described in other types of agriculture [12]. Yet, there are few, if any, biomarkers that can help diagnose this condition sub-clinically.

Several biomarkers in blood or urine have emerged for renal injury, such as serum creatinine and blood urea nitrogen (BUN), which have a long history of clinical use, but these are not particularly sensitive or specific [13]. Proteinuria, or albuminuria, is indicative of late-stage glomerular renal disease, but this would not be particularly useful as an early biomarker of disease.

A novel promising technology for biomarker development comes from extracellular vesicles (EVs) released into the urine by urinary organs, including the kidney and reproductive organs [14] but also from bacteria and large viruses, if these are present [15]. Small EVs (sEVs) typically range less than 200 nm in diameter and include exosomes as well as other types of microvesicles. sEVs carry proteins, miRNAs, mRNAs, and lipids that reflect the state of the cells from which they are released, including disease states [14,16]. While many studies have focused on miRNAs as a valuable source of biomarkers for renal injury [17], other macromolecules, such as lipids, may also be used as biomarkers, especially if the agricultural exposures that these individuals sustain alter lipid biosynthetic pathways and lipid signaling.

For this current study, we used a lipidomics approach to investigate alterations in lipids of urinary EVs as a source of potential novel biomarkers associated with CKDu in sugarcane cutters in Guatemala. Our overarching hypothesis was that lipids in urinary EVs from the sugarcane cutters would be useful as early-detection biomarkers of subclinical kidney disease.

## 2. Materials and Methods

### 2.1. Urine Samples from Male Agricultural Workers in Guatemala

The study population was composed of male sugarcane cutters from Guatemala who were part of a longitudinal study performed through a 6-month harvest season from November 2016 to April 2017. Details of this study, including the study population and environment, are found in Butler-Dawson et al. [18]. Briefly, the study collected clinical and biomarker data before and after a ~10-h work shift at three time points: February, March, and April. For the current study, we utilized urine samples from 15 agricultural workers who were randomly selected from the total study population of 105 workers. Of the 15 workers, 6 were considered controls and -9 were considered to have probable CKDu, defined below. Each worker provided two urine samples, one collected before work and the other after completing a work shift in the fields, in March and/or April 2017. For two individuals with probable CKDu, we had two sets of urine samples. Spot urine samples were collected from workers in sterile cups, placed on ice, and transported to an on-site clinic within one hour after collection. At the clinic, the urine samples were aliquoted into Fisher brand sterile polypropylene tubes without preservatives and frozen at −20 °C. Within one-week, frozen urine aliquots were shipped on dry ice to the University of Colorado Anschutz Medical Campus. Upon arrival, they were immediately stored at −80 °C. Only one worker reported using NSAIDs since waking up and only one worker reported being a current smoker; two workers reported smoking in the past. Glycated hemoglobin (HbA1c) values were low, in the normal range, and all workers had normal blood pressure. The mean age of the workers was 31 years (SD, 8; range, 19–45). The mean body mass index (BMI) for the workers was 22 (SD, 1.2; range, 20–24).

Blood creatinine was measured with handheld point-of-care devices before and after the shift. A threshold of 0.3 mg/dL creatinine was used as a cut-off for acute kidney injury (AKI) based on the Kidney Disease: Improving Global Outcomes (KDIGO) guidelines [19]. The probable CKDu category included those individuals who had an estimated glomerular filtration rate (eGFR) of <90 mL per min per 1.73 m^2^ at all three original study time points. eGFR was calculated based on blood creatinine levels using the CKD-EPI equation [20].

Ethics review and approval for this study were performed by the Colorado Multiple Institutional Review Board (COMIRB # 16-18240) and in Guatemala by the Comite de Etica, Facultad de Medicina, Universidad Francisco Marroquin-Hospital Universitario Esperanza. All participants provided written informed consent at the time of enrollment. The University of Florida (IRB202100528) performed an additional ethics review.

### 2.2. Purification of Extracellular Vesicles

Urine samples were stored frozen at −80 °C at the University of Colorado and shipped to the University of Florida on dry ice for processing. A total of 15 to 20 mL of individual urine samples was centrifuged at 1000× *g* (4 °C) for 15 min to remove particulates. The supernatant was sterile-filtered through 0.22 µm PES Nalgene filters (Thermo Fisher Scientific, Waltham, MA, USA) by vacuum. The purified urine was then transferred into 30 mL polypropylene Optiseal ultra centrifuge tubes (Beckman Coulter, Brea, CA, USA). The tubes were ultracentrifuged at 250,000× *g* for 2 h at 4 °C in a Ti-70 fixed-angle rotor (Beckman Coulter, Brea, CA, USA). All but 1.0 mL of the urine was gently removed with a 5 mL sterile pipette, taking care not to dislodge the off-white-to-transparent pellet. Triple 0.2 µm filtered phosphate-buffered saline (PBS, Corning 21-0404-CV) was added to each tube as a washing step and the solution was subjected to a second round of ultracentrifugation at 250,000× *g* for 2 h at 4 °C. The PBS was removed with a sterile pipette after cutting off the plastic top, taking care not to dislodge the extracellular vesicle pellet. The pelleted vesicles were reconstituted in 200 µL of 3× sterile filtered PBS and removed from the wall with repeated back-and-forth pipetting. The purified small extracellular vesicles (sEVs) were stored in a 1.5 mL low-adhesion microcentrifuge tube (USA Scientific, Ocala, FL, USA) at −80 °C. sEV concentrations were too low for processing for two urine samples of the controls, including one for the AM group and one for the PM group.

### 2.3. Determination of Concentration of the Extracellular Vesicles by Nanoparticle Tracking Analysis

Small extracellular vesicles were stored at −80 °C for 2 to 4 weeks prior to characterization and quantification using the NanoSight NS300 Instrument (Malvern Instruments, Wiltshire, UK), which is available at the Interdisciplinary Center for Biotechnology Research (ICBR). The instrument is equipped with NTA 3.2 software. An automatic infusion pump fed the samples through the machine, with the syringe speed set at 40 µL·s^−1^. This quantified the number and particle size by video analysis of the Brownian motion. The instrument was calibrated with the following parameters: camera type, Scientific Complementary Metal–Oxide–Semiconductor (sCMOS); laser type, Blue488; camera level, 16; slider shutter, 1300; slider gain, 512; number of frames, 1498; temperature, 25 °C. Five 1.0 min videos were captured and analyzed to quantify the average size classes and number of particles. These were typically 1 × 10^8^ to 5 × 10^10^ particles·mL^−1^ resuspension solution and a mean size of 120 nm.

### 2.4. Small Extracellular Vesicle Characterization by Bead-Based Multiplex Flow Cytometry Assay

The surface protein profile of the urine-derived small extracellular vesicles was characterized by a bead-based multiplexing assay using the MACSPlex Human EV IO Kit (ClinMax™ Multiplex Bead Assay Platform for Flow Cytometry; Miltenyi Biotec, Bergisch Gladbach, Germany) according to the manufacturer’s protocol. In brief, 1 × 10^8^ particles of purified sEVs from each individual were diluted to 120 µL using the MACSPlex buffer and then added to each well of the pre-wet filter plate, and 120 µL MACSPlex buffer was used as the blank control. In total, 15 µL of MACSPlex EV Capture beads, a cocktail of fluorescently labeled bead populations coated with specific antibodies binding to thirty-seven EV surface epitopes and two isotype controls, was added to the samples and the blank. The filter plate was incubated overnight at room temperature and protected from light on an orbital shaker (450 rpm). After the incubation, the plate was washed by adding 200 µL MACSPlex buffer per well and centrifuged at 300× *g* for 3 min. Then, 15 µL of MACSPlex EV Detection Reagent cocktail containing allophycocyanin (APC)-conjugated antibodies for CD9, CD63, and CD81 and 135 µL of MACSPlex buffer were added to each well and the plate was incubated at room temperature and protected from light on an orbital shaker (450 rpm) for 1 h, followed by two washes with 200 µL MACSPlex buffer. Samples and the blank control were carefully resuspended in 180 µL MACSPlex buffer and then transferred to 1.5 mL microfuge tubes. The analysis was performed on a CytoFLEX LX Flow Cytometer (Beckman Coulter, Brea, CA, USA) in the Interdisciplinary Center for Biotechnology. The data were acquired and processed by using FlowJo v 10.10.0 (Becton Dickinson & Company, Franklin Lakes, NJ, USA). The relative expression level of each surface protein was determined by the background-subtracted median fluorescence intensity (MFI) and then normalized to the average MFI of CD9, CD63, and CD81 for each sample, with minor correction of single intensities. Markers with MFI values that were below or equal to the MFI of their respective isotope control were excluded from further analysis. The experimental setup and controls, as well as the gating strategy, can be found in the supplementary file. Also included in the supplementary section in Table S1, detailed information based on the MIFlowCyt-EV reporting table template [21] and representative fcs files.

### 2.5. Transmission Electron Microscopy (TEM)

sEVs were analyzed by TEM in the Interdisciplinary Center for Biotechnology, following their standard procedures. In brief, preparations of small extracellular vesicles were transferred to glow-discharged formvar–carbon-coated copper grids and incubated for 5 min. The grids were stained with 1% uranyl acetate for 30 s and, after they had dried, were examined using a FEI Tecnai G2 Spirit Twin Transmission Electron Microscope (Thermo Fisher Scientific, Waltham, MA, USA). Images were acquired with an ultra-scan 2K camera and Digital Micrograph software (version 1.93.1362) (Gatan Inc., Pleasanton, CA, USA).

### 2.6. Lipid Extraction from Small Extracellular Vesicles

Equal numbers of sEVs from the individual urine samples were extracted for lipids using the Bligh and Dyer method [22]. For each urine, 3 × 10^8^ sEVs were used, and each sample was adjusted to 1 mL water with ultrapure analytical-grade water. The samples were placed on ice for 10 min. Afterwards, 2 mL methanol and 0.9 mL methylene chloride were added and the samples were vortexed for 30 s. EquiSplash Lipidomix (Avanti Polar Lipids, Inc., Alabaster, AL, USA) was used as the internal standard and 50 µL at a concentration of 20 μg·mL^−1^ for each lipid was spiked into each sample. EquiSPLASH is a mixture of 13 deuterated lipids including 15:0-18:1(d7) phosphatidylcholine (PC), 18:1(d7) lysophosphatidylcholine (LPC), 15:0-18:1(d7) phosphatidylethanolamine (PE), 18:1(d7) lysophosphatidylethanolamine (LPE), 15:0-18:1(d7) phosphatidylglycerol (PG), 15:0-18:1(d7) phosphatidylinositol (PI), 15:0-18:1(d7) phosphatidylserine (PS), 15:0-18:1(d7)-15:0 triacylglycerol (TAG), 15:0-18:1(d7) diacylglycerol (DAG), 18:1(d7) monoacylglycerol (MAG), 18:1(d7) cholesterol ester (CE), d18:1-18:1(d9) sphingomyelin (SM), and C15 ceramide (CER)-d7. Samples were incubated at room temperature for 30 min and then 1 mL water and 0.9 mL methylene chloride were added to the samples. The samples were mixed gently and then centrifuged at 200× *g* for 10 min. The lower organic phase was carefully removed to a new tube and the process was repeated once by adding 2 mL methylene chloride to the extraction vial, followed by centrifugation. The extracted lipids were dried under a stream of N2 and reconstituted in 50 µL before analysis using LC MS/MS.

### 2.7. LC-MS/MS Conditions

The extracted lipids (5 μL) were injected onto an XBridge Amide 3.5 μm, 4.6 × 150 mm column (Waters, Milford, MA, USA) on an ultra-high performance liquid chromatography system (UHPLC, Shimadzu Co., Kyoto, Japan), which was coupled directly to a QTRAP 6500 mass spectrometer (AB SCIEX, Redwood Shores, CA, USA). The samples were eluted from the column using a binary gradient of acetonitrile: water 95:5 (*v*/*v*) and 50:50 (*v*/*v*) for mobile phases A and B, respectively. Ammonium acetate (1 mM) was added to the mobile phases and the pH was adjusted to 8.2. The linear gradient of solvent B increased to 6% in 6 min and reached 25% within 4 min, 98% within 1 min, and 100% within 2 min, with a flow rate of 0.7 mL·min^−1^. A scheduled multiple reaction monitoring (MRM) method was operated in both negative and positive ion modes, as previously reported [23]. The electrospray ionization source was set to 60 and 80 declustering potential in positive and negative modes, respectively. Collision energy varied from 25 to 60, depending on the lipid species. Other fixed parameters included the following: entrance potential = 10; collision cell exit potential = 15; ion spray voltage = 4.5 kV; and temperature = 300 °C. MultiQuant software (ver 3.0.3) was used to quantify relative concentrations of lipid species that were based on the appropriate internal standard for the class of lipids.

### 2.8. Statistical Analysis

Metaboanalyst (version 6.0) [24,25] was used for statistical analysis and interpretation of lipidomics data. Lipidomics data were normalized by median values, using log transformation and Pareto scaling. The statistical significance of the sEV surface markers was analyzed by ordinary two-way ANOVA with Tukey’s multiple comparisons test (α < 0.05). The expression level of each marker was compared between CKDu and controls and the multiplicity-adjusted p value was reported. The number of particles and the size of each for CKDu and control workers were graphed for morning and after-work collections. These analyses and figures were generated using GraphPad Prism (version 10.3.1).

## 3. Results

### 3.1. Guatemala Participants

Characteristics of the farmworker groups are shown in Table 1. The control (Ctrl) group had an eGFR within normal limits (>90 mL·min^−1^·1.73 m^2−1^), with low serum creatinine levels (<1.2 mg·dL^−1^). However, there was a wide range of changes in creatinine across the workday (0.021 to 0.248 mg·dL^−1^), with three individuals above 0.07 and approaching the cutoff for CKDu. Workers classified as having probable CKDu had eGFR values below the normal limits, had high pre-shift serum creatinine values (>1.5 mg·dL^−1^), and experienced higher changes in creatinine across the workday (>0.1 mg·dL^−1^).

### 3.2. Characterization of Small Extracellular Vesicles

**Figure 1:**
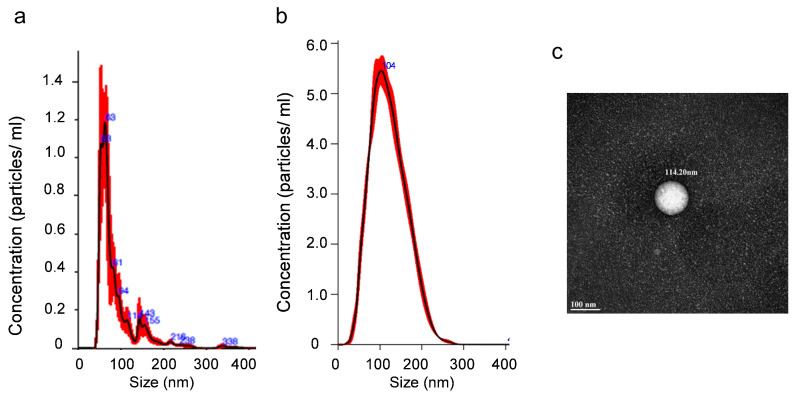
Representative nanoparticle tracking analysis graphs for small extracellular vesicles (sEVs) from (**a**) control (Ctrl) sample and (**b**) possible CKDu sample. sEVs varied in concentration from 5 × 10^9^ mL^−1^ to 5 × 10^10^ mL^−1^. (**c**) Representative TEM image of the sEV preparation for Ctrl, showing a vesicle of 114 nm diameter.

**Figure 2:**
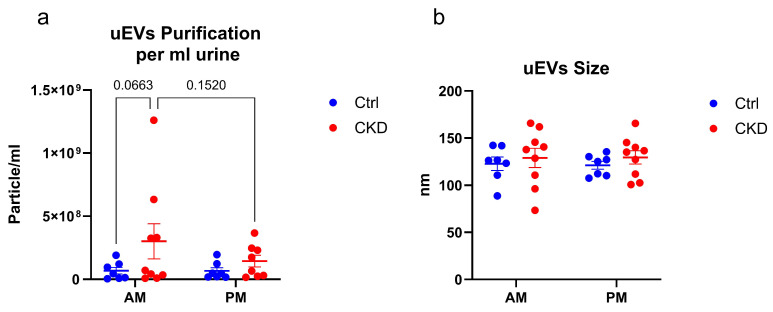
Characterization of small extracellular vesicles (sEVs). (**a**) Particles per ml urine normalized to urinary osmolality. (**b**) Particle size, as determined by nanoparticle tracking analysis (NTA). The concentration and size of sEVs were analyzed by ordinary two-way ANOVA and the comparisons were analyzed by Fisher’s Least Significant Difference (LSD) test. Statistical analyses and the graph were formed by using GraphPad Prism 10.3.1. N= 7 for Ctrl and 9 for CKD.

sEVs were purified from the urine and characterized by NanoSight (Figure 1a,b) and transmission electron microscopy (TEM), as shown for a preparation of Ctrl vesicles (Figure 1c). These images showed the preparations to be free of aggregated proteins and large vesicles and validated the sizes seen by NanoSight. sEV profiles varied for controls and CKDu individuals, with the average number of particles and the variance larger for the CKDu individuals (Figure 2). There was no statistical difference in the diameter of the particles, but the variance in diameter was greater for the CkDu group. There was no measurable difference in number or size over the workday.

**Figure 3:**
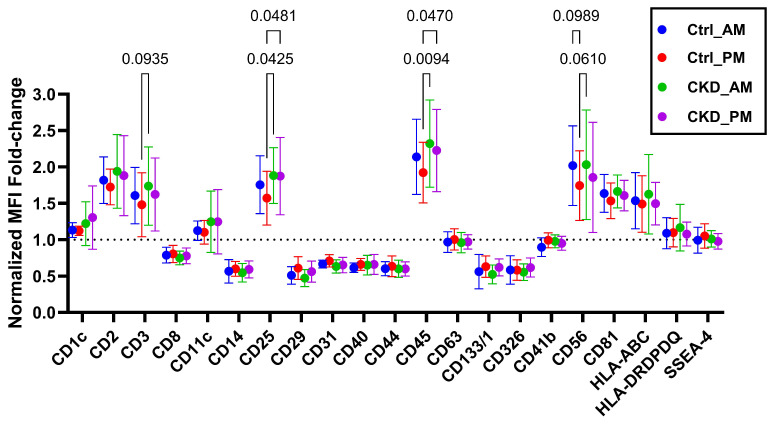
Normalized relative concentrations of CD markers on urinary small extracellular vesicles (sEVs) isolated from agricultural workers in the control (Ctrl) and CKDu (CKD) groups, distinguishing urines collected in the morning and after the work shift for each person. The markers were normalized to the mean values of CD9 + CD81 + CD63, indicated by the dotted line. The normalized expression levels were analyzed by ordinary two-way ANOVA and the comparison of each marker was analyzed by Fisher’s LSD test. Statistical analyses and the graph were formed by using GraphPad Prism 10.3.1.

The sEVs were further characterized for tetraspanins CD9, CD63, and CD81, which are typically present in their membranes, in addition to membrane proteins, using flow cytometry (Figure 3). CD9 values were below the required levels for the method of detection, but CD81 and CD63 were abundant. CD63 expression was about half of that of CD81. The amounts of these two biomarkers were about the same across all the sEVs. Additional membrane proteins that were abundant in the urinary extracellular vesicles were CD1c, CD2, CD3, CD11c, CD25, CD45, CD56, HLA-BC, HLA-DRDPDQ, and SSEA-4. Several of these proteins are associated with the kidney, suggesting the kidney as the originating organ for the sEVs. Interestingly, CD25 and CD45 showed a significant difference between Ctrl and CKDu samples (*p* values < 0.048 and <0.047, respectively, Figure 3).

### 3.3. Lipidomics Analysis

**Figure 4:**
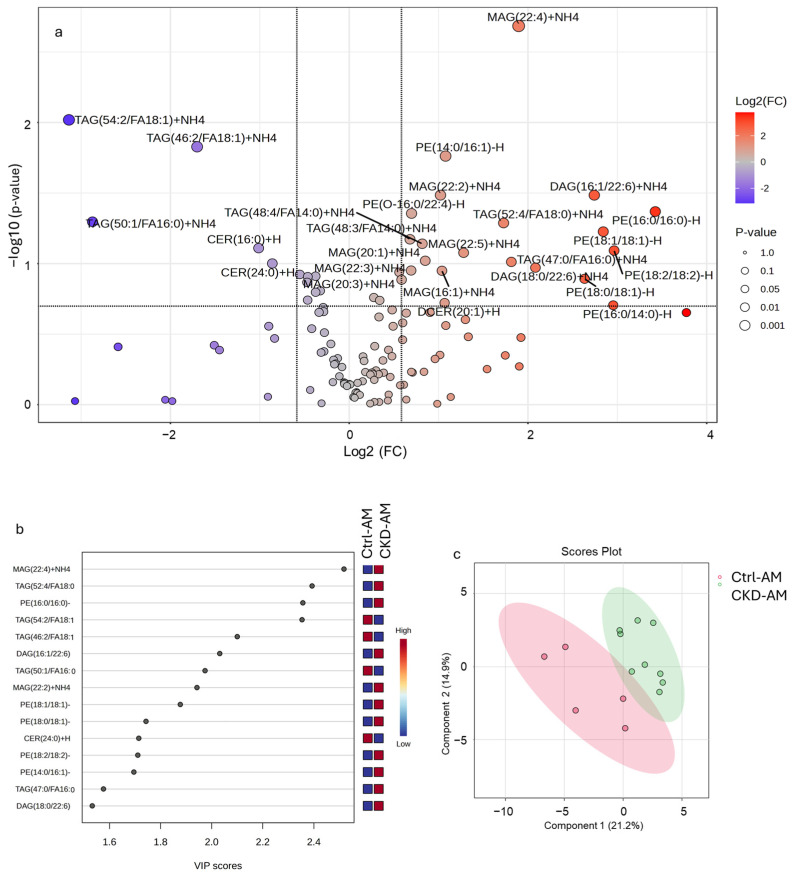
Comparison of lipid expression in small extracellular vesicles (sEVs) isolated from the morning urine of workers classified as controls (Ctrl) and workers classified as possible CKDu. For this analysis, one of the control AM samples was insufficient for analysis and two of the CKDu workers provided urine samples on two dates. (**a**) Volcano plot showing the lipid biomarkers. The diameter of the circles refers to the p-value assigned to the lipids, with lower p values having larger diameters, as shown in the legend on the right. The intensity of the colors is proportional to the log fold change, with more intense colors for higher fold change. (**b**) Top 15 lipid biomarkers that distinguished the two groups based on the variable importance in projection (VIP) score. (**c**) Partial least squares discriminant analysis showing the separation between the two groups. Supplemental Table S2 shows the fold change and p value for each of these lipids. Metaboanalyst software (version 6.0) was used to analyze this data. N is 5 for Ctrl and 9 for CKD.

Equivalent concentrations of sEVs were analyzed for lipid biomarkers. We first compared lipid expression differences between Ctrl AM and CKDu -AM sEVs using Metaboanalyst [24,25]. Figure 4a indicates a volcano plot, showing in red the lipids that were significantly increased and in green the lipids that were significantly decreased in the CKDu group. Figure 4b shows the top 25 lipids that distinguished the groups via variable importance in projection (VIP) scores calculated from the partial least squares discriminant analysis of the two groups. A higher VIP score indicated a greater impact of the metabolite as a discriminant feature among the sample groups. The biomarkers that were identified showed the best discrimination between the Ctrl and CKDu groups. The two groups were well separated from each other, as shown by the partial least squares discriminant analysis (Figure 4c).

**Figure 5:**
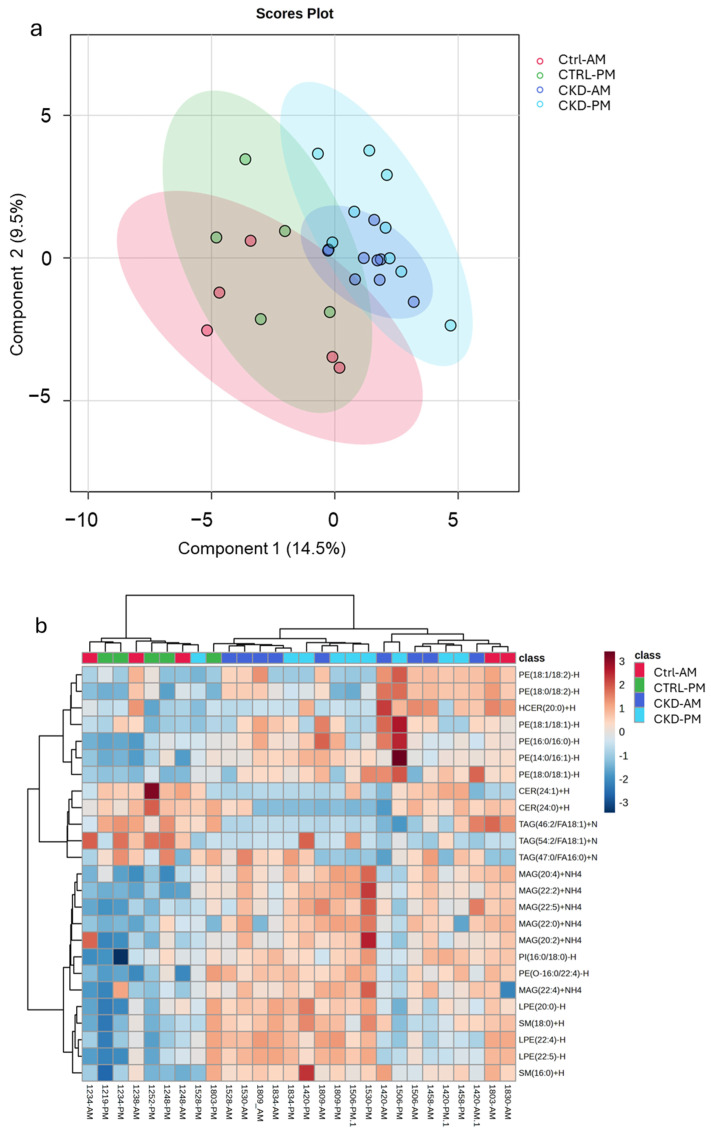
Expression of lipids in small extracellular vesicles (sEVs) isolated from morning and after-shift urine from workers regarded as controls (Ctrl) or possible chronic kidney disease (CKDu). (**a**) Partial least squares discriminant analysis. (**b**) Heat map with top 25 distinguishing lipids. Graphs were formed by Metaboanalyst software (version 6.0) by inputting the lipid concentrations obtained from the targeted lipidomics analysis. N is 5 for Ctrl and 9 for CKD.

In a subsequent analysis, we included sEVs from both the morning and afternoon urines for a partial least squares discriminant analysis. The scores plot (Figure 5a) shows that the lipids from the sEVs isolated from both the morning and afternoon urines from the CKDu group were easily distinguished from the sEVs isolated from the morning urines of controls. However, sEVs isolated from the afternoon urines of the controls seemed to lie at the interface between the morning control group and both CKDu groups. Three of the six workers in the control group experienced a high change in creatinine, and these were the points that appeared closest to the CKDu groups. In fact, if the controls that did not experience a high change in creatinine were removed from the plot, the control workers with the high change in creatinine were remarkably similar to the CKDu groups (Supplementary Figure S1).

A heatmap analysis (Figure 5b) with the top 25 distinguishing lipids shows separation between the control groups and the CKDu groups, with two of the controls appearing intertwined with the CKDu group. These two individuals had relatively high changes in creatinine over the workday (0.124 and 0.144 mg/dL).

**Figure 6:**
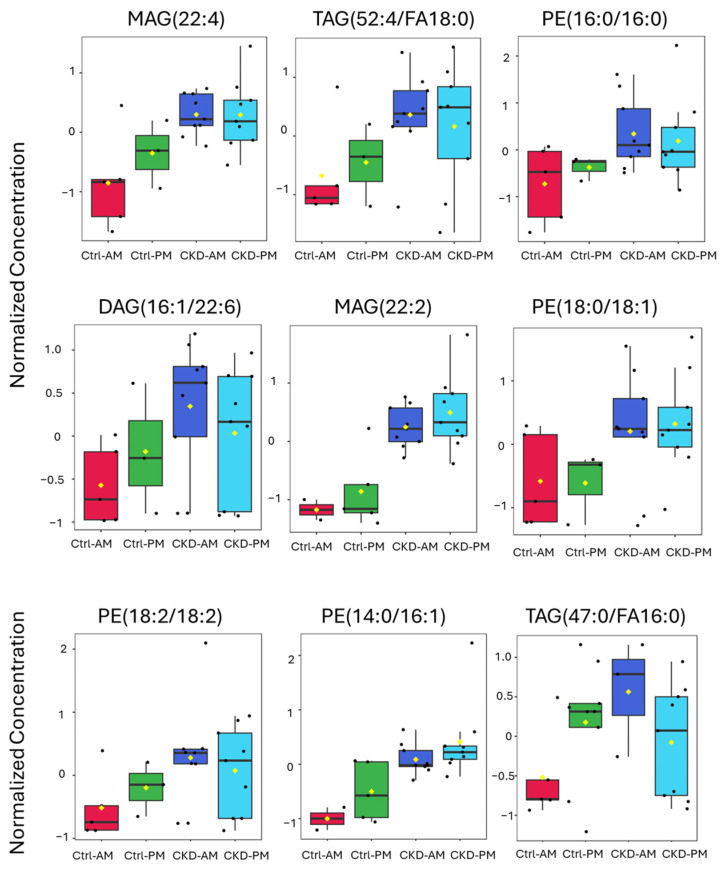
Expression of lipids in small extracellular vesicles (sEVs) isolated from morning and after-shift urine from workers regarded as controls (Ctrl) and workers with possible chronic kidney disease (CKDu), based on the list in the VIP chart in Figure 4b. These lipids increased in the CKDu samples and were predicted to be the most important in explaining the variance between Ctrl and CKDu samples. Lipids are normalized concentrations based on median normalization of log-transformed lipid concentrations using Pareto-scaling across all lipid samples using Metaboanalyst software, (version 6.0).

**Figure 7:**
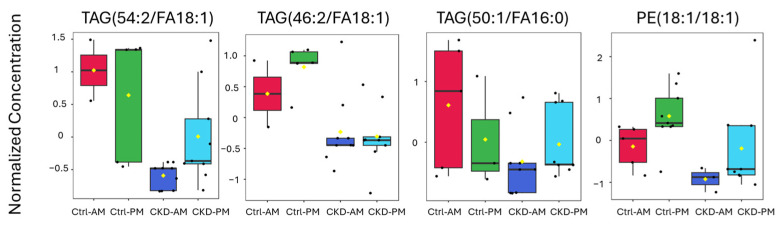
Expression of lipids in small extracellular vesicles (sEVs) isolated from morning and after-shift urine from workers regarded as controls (Ctrl) and workers with possible chronic kidney disease (CKDu), based on the list in the VIP chart in Figure 4b. These lipids decreased in the CKDu samples and were predicted to be the most important in explaining the variance between Ctrl and CKDu samples. Lipids are normalized concentrations based on median normalization of log-transformed lipid concentrations using Pareto-scaling across all lipid samples using Metaboanalyst software (version 6.0).

Following the list of biomarkers in the VIP plot (Figure 4b), we examined the specific expression levels of lipids for each of the groups (Figure 6 and Figure 7). Figure 6 contains lipids that were more highly expressed in the CKDu group than in the controls and Figure 7 contains lipids that were less highly expressed in the CKDu group than in the controls. All the lipids showed a clear difference between the Ctrl-AM group and both the CKDu AM and PM groups. For five of the lipids, including MAG(22:2), PE(16:0/16:0), PE(18:0/18:1), TAG(66:2/FA18:1), and PE(18:1/18:1), the average values for Ctrls (AM and PM) were quite different than both CKDu groups (AM and PM). For other lipids, such as MAG(22:4), TAG(52:4/FA18:0), DAG(16:1/22:6), PE(18:2/18:2), PE(14:0/16:1), TAG(47:0/FA16:0), TAG(54:2/FA18:1), and TAG(50:1/FA16:0), the values for the Ctrl-PM group showed intermediate levels of expression between the Ctrl-AM and both the CKDu AM and PM groups, often grouping close to CKDu samples. These lipids included main membrane constituents such as TAGs, MAGs, and PEs.

**Figure 8:**
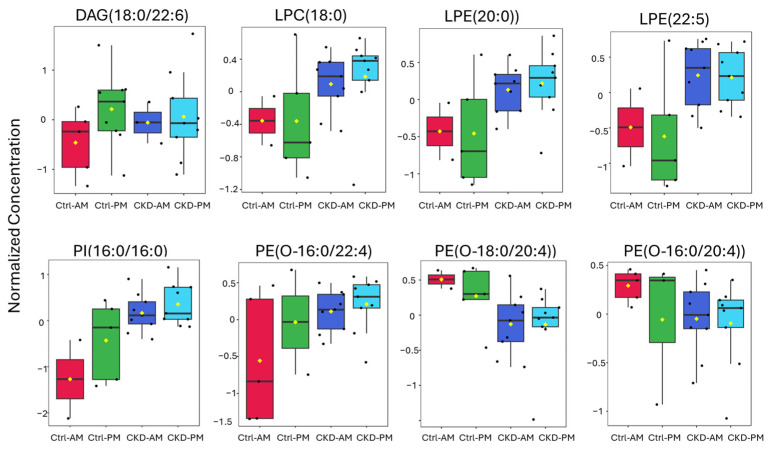
Expression of bioactive lipids in small extracellular vesicles (sEVs) isolated from morning and after-shift urine from workers regarded as controls (Ctrl) and workers with possible chronic kidney disease (CKDu). Lipids are normalized concentrations based on median normalization of log-transformed lipid concentrations using Pareto-scaling across all lipid samples using Metaboanalyst software (version 6.0).

We also plotted the expression levels of bioactive lipids such as DAGs, LPC, LPE, PE(O), and PI (Figure 8). As for the membrane integrity lipids above, there was excellent separation between the Ctrl AM samples and both the CKDu AM and PM samples. Three of the lipids clearly distinguished Ctrl PM samples from the CKDu samples, including LPC(18:0), LPE(20:0), and LPE(22:5). The other five lipids, including DAG(18:0/22:6), PI(16:0/16:0), PE(O-16:0/22:4), PE(O-18:0/20:4), and PE(O-16:0/20:4), were intermediate in expression value between the Ctrl AM samples and both the CKDu AM and PM samples. These lipids appeared to track with early changes in kidney function.

## 4. Discussion

sEVs are double-membrane enveloped vesicles found in all body fluids including urine and, because they contain constituents such as proteins, miRNAs, and lipids from the cells from which they originate, they can be used to diagnose disease states [26,27]. Here, we used urinary sEVs and differential expression of surface proteins and constituent lipid composition as potential biomarkers indicative of kidney disease.

sEVs include exosomes and other microvesicles, such as arrestin domain-containing protein 1-mediated microvesicles (ARMMs) [28,29]. Both types of sEVs are nanometer-scale, cell-derived vesicles in the 30 to 200 nm size range encapsulated by a double bilayer membrane and containing cellular materials, including proteins, mRNAs, and miRNAs [29,30], but differ in their biogenesis. Exosomes are generated in endosomal multivesicular compartments and are secreted when these compartments fuse with the plasma membrane, while ARMMs bud out from the plasma membrane [31]. Both are used for cell–cell communication.

It is clear that precautions must be made for the storage of purified extracellular vesicles, as prolonged storage can degrade their concentration, size, and purity [15,32]. In our study, the sEVs were stored for up to 4 weeks before characterization, a time that has been documented to show minimal effects on protein concentration and the size of EVs [32], and the sEVs we isolated were not used in functional studies. Our main purpose was to see if they could contain biomarkers related to CKDu. The urine samples, however, were stored for 5 years before being shipped to our laboratory. In the Gelibter study [32], plasma storage for 6 months had no statistical effects on protein concentration or the size of particles and size variability. But, we did not study whether storage of the urine could have affected these parameters or the biomarkers we identified. Samples from healthy individuals and those with possible CKDu were treated similarly, but this is a limitation of the present study.

The sEVs we obtained were in the right size range and had prominent levels of tetraspanins CD81 and CD63 and lower levels of CD9. Both CD9 and CD63 have been found to be differentially expressed in the urogenital system and may not be found equally expressed in urinary EVs [33]. In our study, we used the sum of CD81, CD63, and CD9 to normalize the flow cytometry characterization data, and they were all present in the sEVs but in different quantities.

The immune system participates in acute kidney injury (AKI), chronic kidney disease (CKD), and several kidney inflammatory states resulting from transplantation, fibrosis, and other diseases [34,35]. These cells include dendritic cells, natural killer T cells, T and B lymphocytes, neutrophils, and macrophages, all of which contain specific surface markers. Two immune markers, CD25 and CD45, were more highly expressed in sEVs from CKDu than those from controls. These markers are derived from regulatory T cells (Tregs) that are often involved in kidney inflammation [36]. Interestingly, the response of the markers on sEVs from the after-shift urine samples of the controls was like that of the CKDu group for both morning and after-shift samples. This is consistent with initial stages of inflammation caused by the shift.

Other markers of the sEVs, such as CD1c, CD2, CD3, CD11c, CD56, HLA-ABS, and HLA-DRD PDQ, have been detected in kidney tissues and are often elevated due to inflammation or disease, but also have normal roles in kidney function [37,38,39,40,41]. In our study, these markers were similar in all samples, suggesting that, overall, the sEVs originated from similar cells in the kidney. Other surface markers present in the kit are common for sEVs originating from organs other than the kidney [42], and these were below the limit of detection, suggesting the purity of the isolated sEVs and supporting their likely origin.

Protein-based biomarkers associated with damage to different parts of the nephron have been identified [43], including alpha-1 and beta-2 microglobulins, neutrophil gelatinase-associated lipocalin (NGAL), interleukin 18 (IL-18), cystatin-C (Cys-C), trefoil factor 3 (TFF3), kidney injury molecule-1 (KIM-1), clusterin (CLU), and osteopontin (OPN), among others. Of these, KIM-1 appears to be the most predictive of renal injury, followed by CLU, ALB, OPN, and NGAL [44]. However, the utility of these biomarkers for CKDu has been called into question by a study on rats, where urinary KIM-1 and urinary Cys-C were not correlated with the degree of kidney injury [45]. Thus, we had the impetus for our project.

We identified a panel of twenty-one lipids in sEVs that were differentially expressed in CKDu and may be candidates for early detection of CKDu in farmworker populations. When examined as an independent group in the lipidomics workflow, the control PM group tended to be either intermediate in value or close to the possible CKDu group for fourteen of these lipid biomarkers, suggesting that they may be early indicators of kidney injury.

Among the lipid biomarkers that showed the highest discrimination between sEVs from controls and CKDu workers were multiple bioactive lipids with potential functional significance. Three of the biomarkers were plasmalogens PE(O-16:0/22:4), PE(O-18:0/20:4), and PE(O-16:0/20:4), lipids that have a vinyl ether bond at the sn1 position of the glycerol backbone and are normally abundant in membrane lipids. Plasmalogens work in the formation of lipid rafts [28,29,30]. Other bioactive lipids included phosphatidylinositol PI(16:0/16:0), which is an important precursor molecule for phosphoinositides that interact with the PI3K-Akt pathway involved in cell proliferation, survival, and metabolism and may play a role in other forms of chronic kidney disease [46]. Interestingly, this pathway was significantly increased in patients with CKDu resulting from lupus nephritis [37], but it is unknown if this pathway is affected in patients with CKDu.

There were two lysophosphatidylethanolamines, LPE(22:5) and LPE(20:0), which were higher in concentration in sEVs from workers with CKDu than in the controls. These lipids are found in low concentrations in the blood (ranging from 10 to 50 µM [47]) and are made in the membrane when they are needed by the action of lipases. They are bioactive lipids that function as ligands for G-coupled receptors and participate in cell differentiation and immune response [38]. Other studies have shown increases in lysophosphatidylethanolaminesin renal proximal tubule cell injury [40], suggesting these may be biomarkers of renal issues.

One lysophosphatidylcholine, LPC(18:0), was among the bioactive lipids that distinguished controls from CKDu workers. These lipids are made as needed by lipases and are involved in cell signaling and membrane remodeling. Several are known to interact with G-coupled receptors such as GPR4, G2A, and GPR119 [39], involved in inflammation [41]. Several monoacylglycerides also appeared to distinguish the two groups, with some of them showing intermediate values for the after-shift sEVs from controls.

Interestingly, the lipid biomarkers identified in this study correlated well with miRNA biomarkers in the sEVs from the same group of workers (unpublished observation). A combination of miRNA and lipids may work well to distinguish workers in the initial stages of CKDu, well before clinical manifestations of the disease.

Limitations of the current study: The urine samples analyzed were collected in 2016 and 2017 and stored frozen at −80 °C for 5 years before the purification of extracellular vesicles. While there are no studies on the effect of storing urine samples for 5 years on sEV characterization, it is possible that urine storage and sEV storage (up to 4 weeks prior to characterization) altered the size and number of sEVs. A study by Gelibter et al. [32] has shown that storage of EVs for more than 4 weeks after their purification can alter exosome characteristics such as size and number, among others. That study also showed that storage of plasma at −80 °C seemed not to affect these parameters, possibly due to the cryopreservation in the matrix. We do not know if the long-term storage of urine had an effect on EV characterization or lipid oxidation. A study on EV stability in urine showed that storage up to 7 months had no effect on the characterization or abundance of sEVs [48]. sEVs in our study were only thawed once prior to characterization, so they were not exposed to multiple freeze–thaws. Also, urine samples of healthy individuals and those with possible CKDu were treated similarly. The sEVs were not used in functional assays.

The sample group was small, with only six workers considered as controls and six with possible CKDu. A much larger worker group will be necessary to determine if these lipids could be good candidates as early predictors of CKDu. In addition, it would be good to include a group of control individuals who are not farmworkers to eliminate additional confounding variables. Nonetheless, the findings will be informative for future work to determine the efficacy of the biomarkers identified. In addition, a larger group would be useful for constructing a good receiver operating characteristic (ROC) analysis. Workers who develop CKDu are exposed to multiple different stressors that could impact kidney function, including heat, humidity, potential dehydration, agricultural pesticides, silica particles, and others, where sugar cane is grown, so determining the cause(s) of these observed changes in biomarkers in the kidney remains challenging. More studies that assess the interaction between these stressors will be needed to better understand both the individual biomarkers and the disease manifestations.

## 5. Conclusions

We identified a panel of 21 lipids in sEVs that are elevated in workers with possible CKDu and may be candidates for early detection of CKDu in farmworker populations. In addition, two surface protein markers, CD25 and CD45, were differentially expressed in sEVs isolated from farmworkers with CKDu. While this small farmworker study is not definitive and represents a preliminary study, it suggests that lipid biomarkers may be beneficial in assessing workers who may develop CKDu due to occupational hazards in farming. More research will be necessary to validate these biomarkers.

## Figures and Tables

**Table 1:** Participants selected for isolation of extracellular vesicles.

Groups	n	eGFR mL·min^−1^·1.73 m^2−1^	Serum Creatinine (mg·dL^−1^)	Change in Creatinine (mg·dL^−1^)
		Average ± SD	Average ± SD	Average ± SD
Ctrl	6	120 ± 22	0.81 ± 0.21	0.11 ± 0.09
CKDu	6	40 ± 9	2.16 ± 0.40	0.32 ± 0.17

## Data Availability

The dataset is available from the corresponding author upon request.

## Supplementary Materials

The following supporting information can be downloaded at https://www.mdpi.com/article/10.3390/metabo15080523/s1. Table S1: Fold change and p values for phospholipids portrayed as significantly different in the volcano plot in Figure 4a. Table S2: Flow cytometry experiment reporting table. Modified based on MIFlowCyt-EV framework. Figure S1: Partial least squares discriminant analysis of lipid expression in sEVs from control and CKDu workers. For this analysis, controls with a change of less than 0.07 mg/dL creatinine were removed from the afternoon group to see how a substantial change in creatinine affected the lipid expression in sEVs. Flow assay Fcs files: sample files for blanks, two representative controls, and two representative CKD samples for AM and PM collections.

## Abbreviations

The following abbreviations are used in this manuscript:

ACR	Albumin-to-creatinine ratio
AKI	Acute kidney injury
ANOVA	Analysis of variance
APC	Allophycocyanin
ARMMs	Arrestin domain-containing protein 1-mediated microvesicles
BMI	Body mass index
BUN	Blood urea nitrogen
CE	Cholesterol ester
CER	Ceramide
CKD	Chronic kidney disease
CKDu	Chronic kidney disease of unknown etiology
CLU	Clusterin
COMIRB	Colorado Multiple Institution Review Board
Ctrl	Control
Cyc-C	Cystatin-C
DAG	Diacylglycerol
eGFR	Estimated glomerular filtration rate
EVs	Extracellular vesicles
Fcs	Standard files used to store flow cytometry data
HbA1c	Glycated hemoglobin
IL-18	Interleukin 18
IRB	Institutional Review Board
KDIGO	Kidney Disease: Improving Global Outcomes
KIM-1	Kidney injury molecule-1
LC MS/MS	Liquid chromatography tandem mass spectrometry
LPC	Lysophosphatidylcholine
LPE	Lysophosphatidylethanolamine
LSD	Least significant difference
MACSPlex	ClinMax™ Multiplex Bead Assay Platform for Flow Cytometry
MAG	Monoacylglycerol
MFI	Median fluorescence intensity
MRM	Multiple reaction monitoring
NGAL	Neutrophil gelatinase-associated lipocalin
NSAIDS	Nonsteroidal anti-inflammatory drugs
NTA	Nanoparticle tracking analysis
OPN	Osteopontin
PBS	Phosphate-buffered saline
PC	Phosphatidylcholine
PE	Phosphatidylethanolamine
PE(O)	Phosphatidylethanolamine plasmalogen
PG	Phosphatidylglycerol
PI	Phosphatidylinositol
PS	Phosphatidylserine
ROC	Receiver operating characteristic
sCMOS	Scientific Complementary Metal–Oxide–Semiconductor
sEV	Small extracellular vesicle
SM	Sphingomyelin
TAG	Triacylglycerol
TEM	Transmission electron microscopy
TFF3	Trefoil factor 3
VIP	Variable importance in projection
