# Supplementary material for: Novel Lipid Biomarkers of Chronic Kidney Disease of Unknown Etiology Based on Urinary Small Extracellular Vesicles: A Pilot Study of Sugar Cane Workers
Source: Metabolites. 2025 Aug 2;15(8):523. doi: 10.3390/metabo15080523 (PMC12388243; doi:10.3390/metabo15080523)
Supplement: Supplementary file 1 [file metabolites-15-00523-s001.zip › metabolites-3712452-supplementary.pdf]

Supplementary Information

Novel lipid biomarkers of chronic kidney disease of unknown etiology based on urinary small extracellular vesicles from a pilot study of sugar cane workers

Jie Zhou[1], Kevin J. Kroll[2], Jaime Butler-Dawson[3], Lyndsay Krisher[4], Abdel A. Alli[5], Chris Vulpe[6], Nancy D. Denslow[7*]

1 Department of Physiological Sciences and Center for Environmental and Human Toxicology, University of Florida, Gainesville, FL, jiezhou68@ufl.edu
2 Department of Physiological Sciences and Center for Environmental and Human Toxicology, University of Florida, Gainesville, FL, krollk@ufl.edu
3 Department of Environmental and Occupational Health, Colorado School of Public Health, University of Colorado Anschutz Campus, Aurora, Colorado, USA, jaime.butler-dawson@cuanschutz.edu
4 Department of Environmental and Occupational Health, Colorado School of Public Health, University of Colorado Anschutz Campus, Aurora, Colorado, USA, lyndsay.krisher@cuanschutz.edu
5 Department of Medicine Division of Nephrology, Hypertension, and Renal Transplantation and De-partment of Physiology and Aging, University of Florida College of Medicine, Gainesville Fl, aal-li@ufl.edu
6 Department of Physiological Sciences and Center for Environmental and Human Toxicology, University of Florida, Gainesville, FL, cvulpe@ufl.edu
7 Department of Physiological Sciences and Center for Environmental and Human Toxicology, University of Florida, Gainesville, FL, ndenslow@ufl.edu

* Correspondence: ndenslow@ufl.edu; Tel.: +1 352-294-4642

Supplemental Table S1. Flow Cytometry Experiment Reporting Table. Modified based on MIFlowCyt-EV framework

**Framework Criteria**	
1. Preanalytical variables conforming to MISEV guidelines.	EV samples were isolated from human urine samples by ultra centrification. Purifed samples were suspended in filtered PBS and stored at -80C until the experiment.
2. Experimental design according to MIFlowCyt guidelines.	1.1 **Aim**: To detect the expression level of 37 EV surface epitopes in urinary EVs from farmworkers between individuals with impaired kidney functions and healthy controls by using a beads-binding flow cytometry assay. We hypothesize that individuals with impaired kidney functions will have higher level of kidney disease associated markers. 1.2 **Keywords**: EV; CKD. 1.3 **Experimental variables**: Urinary EV samples were purified from 17 individuals with impaired kidney functions and healthy controls. There was no significant difference in age, sex, blood-pressure between these individuals.
3.1. Sample staining details	All samples were resuspended in MACSPlex buffer, then incubated overnight with a cocktail of antibody-binding fluoresce labeled beads. On the next day, samples were further stained with MACSPlex EV Detection Reagent (APC-conjugated antibodies).
3.2. Sample washing details	200 ul of MACSPlex buffer per sample well was used during all three washing steps. All samples including the blank were washed one time after the initial overnight staining and washing two times after the 2nd APC-conjugated staining.
4.1. EV diameter /surface area/ volume approximation	EV diameter was measured by NanoSight NS300. All examined samples had similar size range.
4.2. EV number/ concentration.	1.00E+08 particles of purified extracellular vesicle of each sample, based on the concentration measured by NanoSight NS300.
4.3. Sample dilution details	1.00E+08 particles of purified extracellular vesicle of each sample were diluted to 120 µl by MACSPlex buffer.
5.1. Buffer with reagent controls.	120 ul MACSPlex buffer was used as the blank control. MFI value was used for background calculation.
5.2. Unstained controls.	N/A
5.3. Isotype controls.	Recombinant human IgG1 and mouse IgG1 were used as isotype controls. All antibody-binding beads and isotype-binding beads were manufactured by Miltenyi biotec (Cat# 130-108-813).
5.4. Single-stained controls.	N/A
5.5. Procedural controls.	Pre-stained setup beads from the kit were used.

6.1. Trigger Channel(s) and Threshold(s).	PE, PITC and APC channels.
6.2. Flow Rate / Volumetric quantification.	Flow rate 30-60 ul per minute. 10,000 events were recorded.
6.3. Fluorescence/ Light Scatter Calibration.	Daily fluorescence and light scatter calibrations were performed by the core facility.

Supplemental Table S2. Fold change and p values for phospholipids portrayed as significantly different in the volcano plot in Fig. 3a.

	FC	log2(FC)	raw.pval	-LOG10(p)
MAG(22:4)+NH4	3.3659	1.751	0.0028422	2.5463
TAG(54:2/FA18:1)+NH4	0.072276	-3.7903	0.0061719	2.2096
TAG(46:2/FA18:1)+NH4	0.29853	-1.744	0.014996	1.824
PE(14:0/16:1)-H	2.021	1.0151	0.022051	1.6566
PE(16:0/16:0)-H	10.765	3.4283	0.031076	1.5076
TAG(52:4/FA18:0)+NH4	3.6863	1.8822	0.033032	1.4811
DAG(16:1/22:6)+NH4	6.6941	2.7429	0.035202	1.4534
MAG(22:2)+NH4	1.9036	0.92876	0.037663	1.4241
TAG(50:1/FA16:0)+NH4	0.12971	-2.9467	0.050117	1.3
PE(O-16:0/22:4)-H	1.5503	0.63257	0.051425	1.2888
CER(16:0)+H	0.49448	-1.016	0.061778	1.2092
PE(18:1/18:1)-H	6.8561	2.7774	0.063229	1.1991
TAG(48:3/FA14:0)+NH4	1.5413	0.62418	0.077524	1.1106
PE(18:2/18:2)-H	7.9273	2.9868	0.080479	1.0943
PE(O-16:0/20:4)-H	0.75169	-0.41179	0.085833	1.0663
TAG(48:4/FA14:0)+NH4	1.6807	0.74908	0.087996	1.0555
PE(O-16:0/20:3)-H	0.70721	-0.49978	0.094321	1.0254
CER(24:0)+H	0.53845	-0.89311	0.095434	1.0203
MAG(22:5)+NH4	2.352	1.2339	0.095596	1.0196
LPE(20:3)-H	0.66171	-0.59572	0.097165	1.0125
PE(O-16:0/20:5)-H	0.70574	-0.5028	0.10176	0.9924
TAG(47:0/FA16:0)+NH4	3.4716	1.7956	0.10182	0.99218
MAG(20:1)+NH4	1.7437	0.80217	0.10848	0.96466
DAG(18:0/22:6)+NH4	4.1966	2.0692	0.11718	0.93115
MAG(22:3)+NH4	1.5459	0.62842	0.12437	0.90527
MAG(16:1)+NH4	1.9744	0.98138	0.12651	0.89786
LPE(20:5)-H	0.74823	-0.41844	0.12728	0.89522
MAG(20:4)+NH4	1.4049	0.49049	0.1301	0.88572
PE(18:0/18:1)-H	6.924	2.7916	0.13391	0.87319
PE(O-18:0/20:4)-H	0.71114	-0.4918	0.14122	0.85009
MAG(20:3)+NH4	1.4235	0.5094	0.14983	0.82439
PE(16:0/14:0)-H	9.3011	3.2174	0.19526	0.70939

Supplemental Figure S1

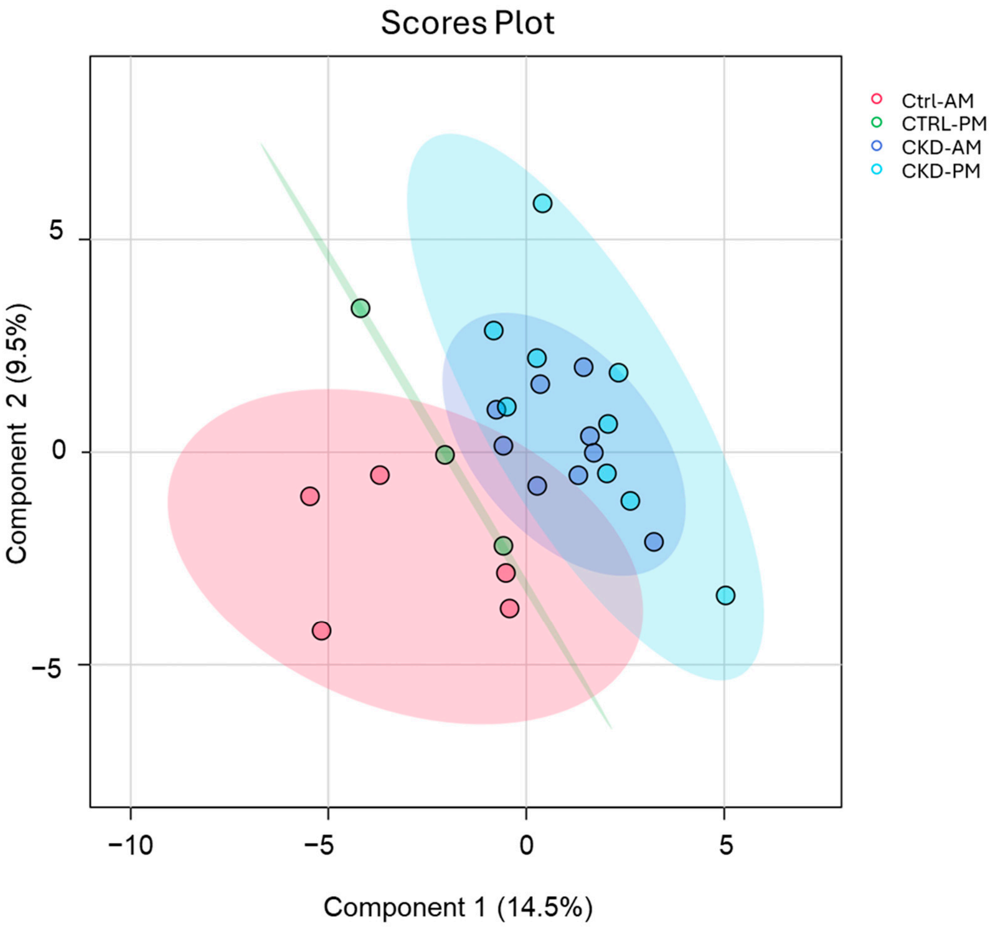

Suppl. Figure S1. Partial least squares discriminant analysis showing the expression of lipids in small extracellular vesicles isolated from morning and after-shift urines from workers regarded as Controls (C), or possible chronic kidney disease (CKDu). For this graph, the C-PM group contained only workers that experienced a large shift in creatinine across the workday.

Representative Flow Assay FCS files:

a) Blank.fcs
b) Sample 1 Ctrl 1219 AM.fcs
c) Sample 2 Ctrl 1219 PM.fcs
d) Sample 3 Ctrl 1234 AM.fcs
e) Sample 4 Ctrl 1234 PM.fcs
f) Sample 15 CKD 1834 AM.fcs
g) Sample 16 CKD 1834 PM.fcs
h) Sample 31 CKD 1809 AM.fcs
i) Sample 32 CKD 1809 PM.fcs
j) Setup beads.fcs
